# Primary and Immortalized Human Respiratory Cells Display Different Patterns of Cytotoxicity and Cytokine Release upon Exposure to Deoxynivalenol, Nivalenol and Fusarenon-X

**DOI:** 10.3390/toxins9110337

**Published:** 2017-10-25

**Authors:** Silvia Ferreira Lopes, Gaëlle Vacher, Eleonora Ciarlo, Dessislava Savova-Bianchi, Thierry Roger, Hélène Niculita-Hirzel

**Affiliations:** 1 Service of Occupational Hygiene, Institute for Work and Health (IST), University of Lausanne and Geneva, Epalinges 1066, Switzerland; silvia.ferreira@unil.ch (S.F.L.); gaelle.vacher@u-bordeaux.fr (G.V.); Dessislava.Savova-Bianchi@chuv.ch (D.S.-B.); 2 Infectious Diseases Service, Lausanne University Hospital, Epalinges 1066, Switzerland; Eleonora.Ciarlo@chuv.ch (E.C.); Thierry.Roger@chuv.ch (T.R.)

**Keywords:** mycotoxins, deoxynivalenol, nivalenol, fusarenon, airway epithelial cells, cytotoxicity, cytokine

## Abstract

The type B trichothecene mycotoxins deoxynivalenol (DON), nivalenol (NIV) and fusarenon-X (FX) are structurally related secondary metabolites frequently produced by *Fusarium* on wheat. Consequently, DON, NIV and FX contaminate wheat dusts, exposing grain workers to toxins by inhalation. Those trichothecenes at low, relevant, exposition concentrations have differential effects on intestinal cells, but whether such differences exist with respiratory cells is mostly unknown, while it is required to assess the combined risk of exposure to mycotoxins. The goal of the present study was to compare the effects of DON, NIV and FX alone or in combination on the viability and IL-6 and IL-8-inducing capacity of human epithelial cells representative of the respiratory tract: primary human airway epithelial cells of nasal (hAECN) and bronchial (hAECB) origin, and immortalized human bronchial (16HBE14o-) and alveolar (A549) epithelial cell lines. We report that A549 cells are particularly resistant to the cytotoxic effects of mycotoxins. FX is more toxic than DON and NIV for all epithelial cell types. Nasal and bronchial primary cells are more sensitive than bronchial and alveolar cell lines to combined mycotoxin mixtures at low concentrations, although they are less sensitive to mycotoxins alone. Interactions between mycotoxins at low concentrations are rarely additive and are observed only for DON/NIV and NIV/FX on hAECB cells and DON/NIV/FX on A549 cells. Most interactions at low mycotoxin concentrations are synergistic, antagonistic interactions being observed only for DON/FX on hAECB, DON/NIV on 16HBE14o- and NIV/FX on A549 cells. DON, NIV and FX induce, albeit at different levels, IL-6 and IL-8 release by all cell types. However, NIV and FX at concentrations of low cytotoxicity induce IL-6 release by hAECB and A549 cells, and IL-8 release by hAECN cells. Overall, these data suggest that combined exposure to mycotoxins at low concentrations have a stronger effect on primary nasal epithelial cells than on bronchial epithelial cells and activate different inflammatory pathways. This information is particularly relevant for future studies about the hazard of occupational exposure to mycotoxins by inhalation and its impact on the respiratory tract.

## 1. Introduction

Type B trichothecene such as deoxynivalenol (DON), nivalenol (NIV) and fusarenon-X (FX) are found in the spores and hyphae of the *Fusarium* species responsible for Fusarium head blight disease, such as *F. graminearum* and *F. culmorum*. Those phytophathogenic fungi infect the wheat at the outside of the grain head and move inward. As a result, mycotoxins are found in larger quantities in the outer layers of the kernel and the chaff than in the kernels themselves [1,2]. During grain or straw wheat handling, *Fusarium* hyphae fragments and spores are aerosolized with their toxin content and a large proportion have a size small enough to be inhaled [3].

While *F. graminearum* and *F. culmorum* produce a fairly large number of secondary metabolites with more or less known toxic effects, DON and NIV are among the most prevalent mycotoxins occurring in wheat samples analyzed in recent years all over the world [4,5]. Some isolates of *Fusarium* might convert DON in NIV, although others might convert NIV to FX [6]. Air samples collected during grain unloading contain DON at mean airborne concentrations and maximum concentrations of 37 ng/m^3^ and 2.59 μg/m^3^ in Canadian grain elevators [7] and 53 ng/m^3^ and 121 ng/m^3^ in Swiss grain elevators [3]. Similar DON concentrations have been measured in Finnish farms during cattle feeding, grain drying and milling [8] as well as during wheat threshing [3]. NIV at mean airborne concentrations and maximum concentrations of 46 ng/m^3^ and 297 ng/m^3^ have been described in Swiss grain elevators during grain unloading [3]. While other mycotoxins are present in aerosols generated during grain handling [9] and in settled grain dusts [2,10], FX has not been detected in settled grain dust [10], but in cereals along with DON and NIV [11,12].

Occupational studies in Europe have reported higher levels of urinary biomarkers of DON (DON and DOM-1, i.e., deepoxy-deoxynivalenol) in active farmers exposed to grain dusts than in retired farmers exposed to mycotoxins through diet [13]. This difference was linked to massive exposure to mycotoxins of farmers working in a confined area with grain contaminated by *F. graminearum*. Massive exposure to fungal contaminated agricultural materials has been associated to the development of an acute, febrile pattern of respiratory illness called organic dust toxic syndrome (ODTS). However, there is still uncertainty regarding the etiologic agents responsible for this illness since grain dusts are a complex matrix containing not only fungi and fungal mycotoxins but also bacteria and bacterial endotoxins. Therefore, neither OSHA (Occupational Safety and Health Administration), NIOSH (National Institute for Occupational Safety and Health) or ACGIH (American Conference of Governmental Industrial Hygienists) have proposed occupational respiratory exposure limit values for specific mycotoxins, but for organic dusts. Chronic exposure to mycotoxins might drive chronic illnesses. A longitudinal survey of farmers over more than two decades suggests a relationship between grain farming and mid-pregnancy deliveries in farmer families, possibly linked to mycotoxin exposure [14]. Small increase relative risks were observed in several cancers in female but not in male farmers [15]. Consequently, the potential risk of occupational exposure to mycotoxins has been formally recognized [16].

To assess the combined risk of exposure to structurally related toxins, a toxic equivalency factor (TEF) is supposed to be defined [17,18,19]. However, applying TEFs to DON, NIV and FX would assume that these mycotoxins have similar modes of action and pathogenic effects. Yet increasing amounts of evidence suggest that this may not be the case. Indeed, while DON, NIV and FX bind eukaryotic ribosomes and inhibit protein synthesis [20] and modulate cytokine expression [20,21,22], the toxicity of DON on intestinal cells significantly deviates from that of NIV [23] and FX [24,25,26,27]. Dissimilarities of action of DON, NIV and FX might also occur in lung tissues. Although DON absorption was associated with the up-regulation of interleukin (IL)-1β, IL-6 and tumor necrosis factor (TNF) mRNA in the lungs, spleen and liver [28], inhibition of protein synthesis, and proliferation and survival rate reduction of the human lung adenocarcinoma A549 cell line [29], DON affected IL-6 and IL-8 release by A549 cells, but not by the bronchial epithelial BEAS-2B cell line [30].

The aim of the present study was to compare the toxicity and the cytokine-inducing capacity of DON, NIV and FX on human airway cells from different regions of the respiratory tract considering that depending on the size, shape and aggregation with other substances, airborne dust particles deposit from the nasopharyngeal area to the lung alveoli [31]. Moreover, the effects of single, binary and ternary combinations of DON, NIV and FX have been assessed in order to define the type of interactions between mycotoxins that naturally co-occur in inhalable fractions of grain dusts. Indeed, in mixture toxicology, the non-interaction or additivity assumption might be applied if several chemicals act in concert without interference. However factors, such as toxicokinetic interactions, might lead to deviations from expected additive effects, indicative of synergisms or antagonisms [32,33]. Finally, considering that immortalized cell lines and primary cells may react differently to mycotoxins [34], experiments were performed with the immortalized human alveolar A549 and bronchial 16HBE14o-cell lines as well as with primary human airway epithelial cell lines of nasal (hAECN) and bronchial (hAECB) origin.

## 2. Results

### 2.1. Cytotoxicity of DON, NIV and FX on Immortalized and Primary Airway Cell Lines

**Figure 1:**
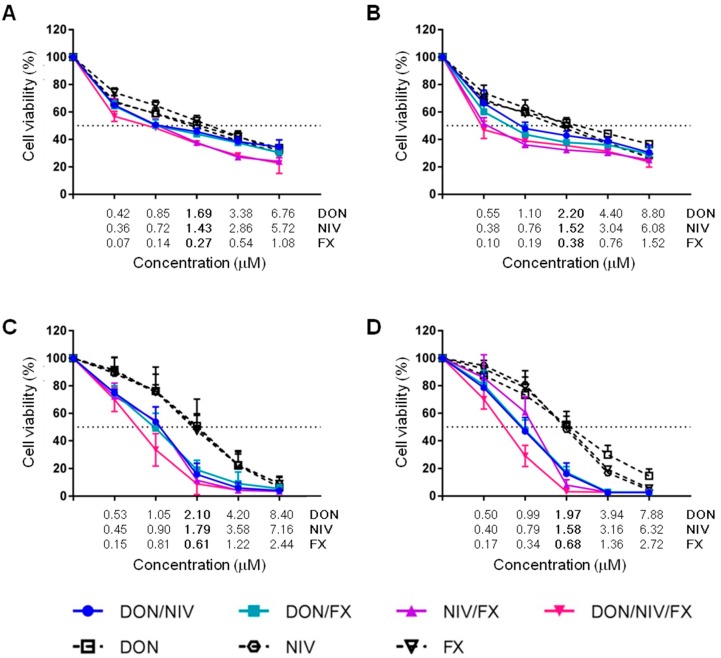
Cytotoxic effects of DON, NIV and FX alone or in combination on A549 (**A**), 16HBE14o- (**B**), hAECN (**C**) and hAECB (**D**) cells. Data, calculated as described in *Materials and Methods*, are median ± SD of three independent experiments performed in triplicate.

An MTT cytotoxicity assay based on mitochondrial activity was used to compare the cytotoxicity of increasing concentrations of DON, NIV and FX on immortalized human alveolar (A549) and bronchial (16HBE14o-) epithelial cell lines and primary human airway epithelial cells of nasal (hAECN) and bronchial (hAECB) origin. Preliminary experiments revealed that A549 cells were particularly resistant to mycotoxins. Following 24 h of incubation, the growth of A549 cells was weakly inhibited, while the growth of 16HBE14o-, hAECN and hAECB cells was inhibited by at least 50%. Thus, in subsequent experiments, A549 cells were incubated with mycotoxins for 48 h while 16HBE14o-, hAECN and hAECB cells were incubated with mycotoxins for 24 h. Under these conditions, DON, NIV and FX were cytotoxic for the four cell types in a dose-dependent manner, allowing the calculation of the IC_50_ reported in Table 1. The IC_50_ of DON (1.69–2.2 μM) and NIV (1.43–1.79 μM) for A549, 16HBE14o-, hAECB and hAECN cells were not significantly different. FX (IC_50_ = 0.27–0.61 μM) was more cytotoxic than DON and NIV for all cell types, and more cytotoxic for A549 and 16HBE14o-cell lines (IC_50_ = 0.27 ± 0.08 and 0.38 ± 0.06 μM) than for hAECB and hAECN cell lines (IC_50_ = 0.68 ± 0.14 and 0.61 ± 0.12 μM; *p* > 0.05). Interestingly although established cell lines were more sensitive than primary cell lines to low mycotoxin concentrations, they were much more resistant to high mycotoxin concentrations (Figure 1, Table 2).

### 2.2. Cytotoxicity of Binary and Ternary Combinations of DON, NIV and FX on Established and Primary Airway Cell Lines

**Figure 2:**
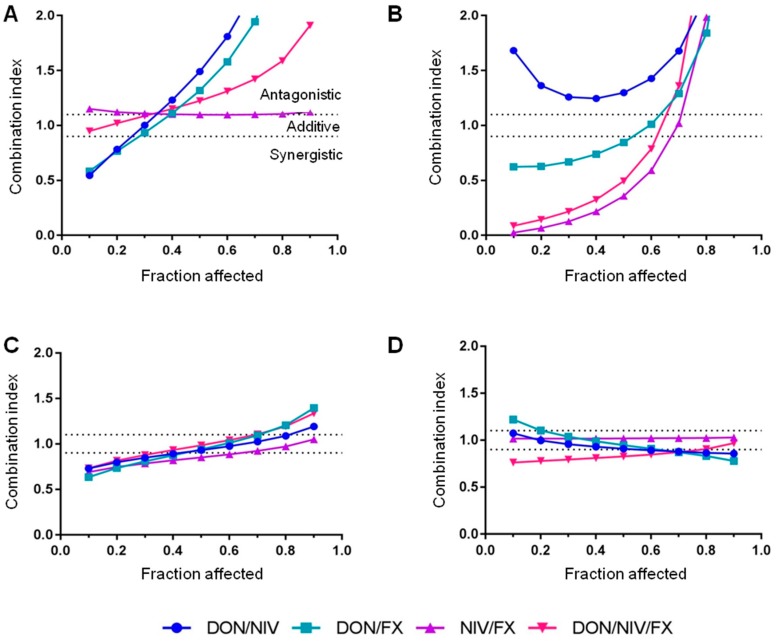
Combination index (CI) versus cell fraction affected curves for binary and ternary mixtures of DON, NIV and FX on A549 (**A**), 16HBE14o- (**B**), hAECN (**C**) and hAECB (**D**) cells. CI < 0.9, CI = 0.9–1.1 and CI > 1.1 reveal synergistic, additive and antagonistic effects, respectively. Data are derived from three independent experiments performed in triplicate.

Mixtures of two or three mycotoxins were applied to A549, 16HBE14o-, hAECN and hAECB cells at concentrations affecting similar cell fractions (i.e., similar f_a_, fraction affected) to assess the interactions between DON, NIV and FX. Binary and ternary combinations increased cytotoxicity in a dose-dependent manner (Figure 1). When compared to the effects of single mycotoxins, combinations of two mycotoxins decreased the viability of A549 and 16HBE14o immortalized cell lines, but more robustly that of hAECN and hAECB primary cell lines. Cytotoxic effects were slightly more pronounced when using the ternary combination on primary cell lines than on immortalized cell lines (Figure 1). The interactions between DON, NIV and FX were determined by calculating the combination index (CI) at various cytotoxic levels, i.e., at f_a_ ranging from 10 to 90% (Figure 2). CI < 0.9, CI = 0.9–1.1 and CI > 1.1 reveal synergistic, additive and antagonistic effects, respectively. CI/f_a_ curves differed greatly depending on mycotoxin combinations, mycotoxin concentrations and cell types, and differed more for immortalized cell lines than for primary cell lines (Figure 2). At low concentrations, DON, NIV and FX interacted in a synergistic way for all combinations tested only in hAECN cells. At these concentrations, synergistic interactions were observed for all combinations with FX in 16HBE14o-cells, for DON/NIV and DON/FX in A549 cells and for ternary combination in hAECB cells. Note the additive interactions between DON/NIV and NIV/FX on hAECB primary cell lines, but not in 16HBE14o-immortalized cell lines. DON/FX interacted in an antagonistic way only in hAECB cells.

**Figure 3:**
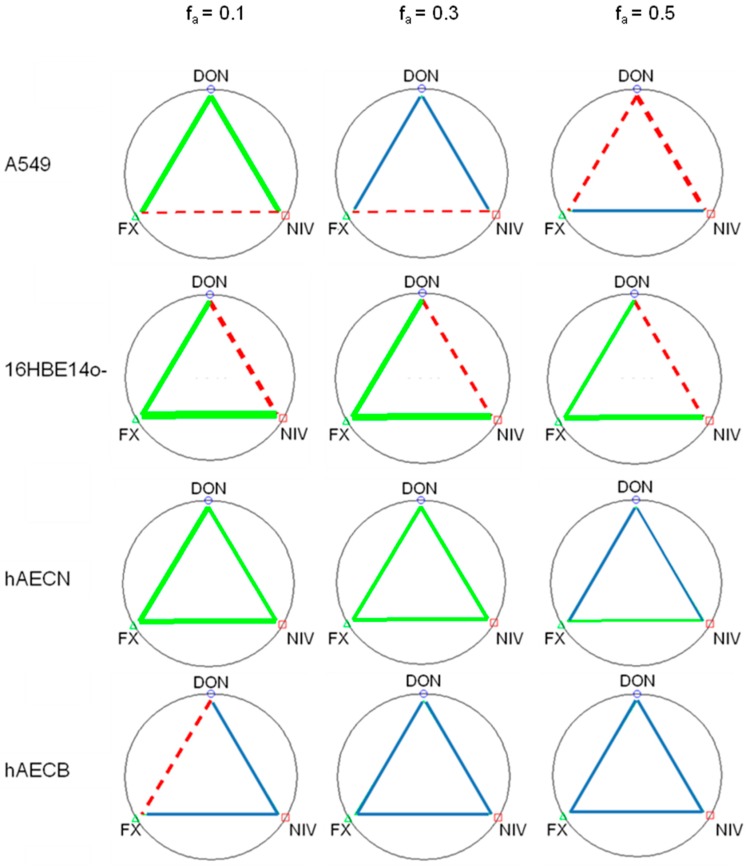
Polygonographs showing the interactions between DON, NIV and FX at doses affecting the viability of 10%, 30% and 50% of cells (respectively f_a_ = 0.1, 0.3 and 0.5). Blue, green and red lines depict additive, synergistic and antagonistic effects, respectively. Line thickness is proportional to the interaction degree. Data are derived from three independent experiments performed in triplicate.

In order to characterize the interactions between mycotoxins at doses lower than IC_50_, binary interactions at concentrations of DON, NIV and FX affecting 10%, 30% and 50% of cell viability (i.e., f_a_ = 0.1, 0.3 and 0.5) were depicted as polygonographs (Figure 3). The most complex picture was observed for A549 cells, while more conserved interactions were observed for 16HBE14o-, hAECN and hAECB cells. For A549 cells, DON/FX and DON/NIV combinations at f_a_ 0.1, 0.3 and 0.5 resulted in synergistic, additive and antagonistic effects, respectively. FX/NIV had antagonistic effects at f_a_ 0.1 and 0.3, but an additive effect at f_a_ 0.5. For 16HBE14o-cells, DON/FX and FX/NIV had synergistic effects while DON/NIV had an antagonistic effect at f_a_ 0.1, 0.3 and 0.5. For hAECN cells, DON/FX, DON/NIV and FX/NIV had synergistic effects at f_a_ 0.1 and 0.3. At f_a_ 0.5, FX/NIV had a weak synergistic effect while DON/FX and DON/NIV had additive effects. For hAECB cells, DON/FX had an antagonistic effect at f_a_ 0.1 while all other mycotoxin combinations tested revealed weak additive effects. DON/FX and NIV/FX interactions were closest between 16HBE14o- and hAECN cell lines.

### 2.3. IL-6 and IL-8 Release by Immortalized and Primary Airway Cell Lines Exposed to DON, NIV and FX

**Figure 4:**
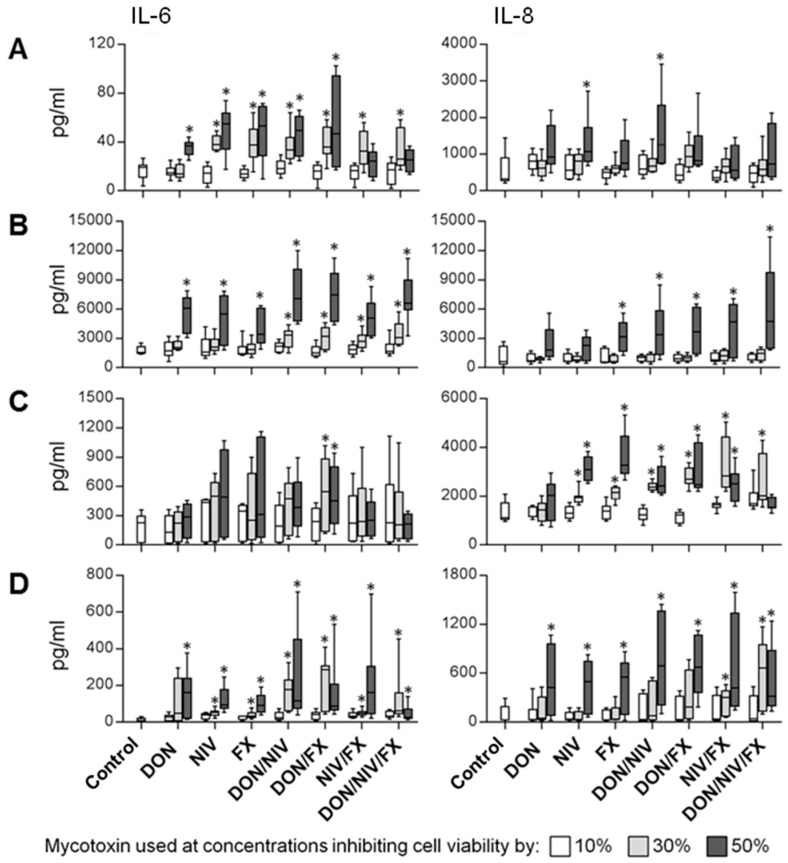
Box plots (min to max) showing IL-6 (left) and IL-8 (right) concentrations in cell culture supernatants of A549 (**A**), 16HBE14o- (**B**), hAECN (**C**) and hAECB (**D**) cells exposed for 24 h (16HBE14o-, hAECN and hAECB) or 48 h (A549) to single and combinational mixtures of DON, NIV and FX. Bar colors indicate the concentrations at which mycotoxins were used: white, light grey and dark grey for concentrations affecting the viability of 10%, 30% and 50% of cells, respectively. Data are from three independent experiments performed in triplicate. * Indicates statistically significant differences (*p* < 0.05) in cytokine levels between cells exposed and not exposed to mycotoxins.

Inflammation is a hallmark of the toxicity of DON and FX on epithelial intestinal cells [24]. To address whether mycotoxins induced pro-inflammatory responses by airway cells, we measured the concentrations of IL-6 and IL-8 in culture supernatants of cells exposed for 24 h (16HBE14o-, hAECN and hAECB cells) or 48 h (A549 cells) to medium or DON, NIV and FX used alone or in combination at f_a_ 0.1, 0.3 and 0.5 (Figure 4). All cell types produced IL-6 and IL-8 at baseline and upon mycotoxin exposure, albeit at different levels (ranking of order for IL-6 16HBE14o->>hEACN>>hAECB>A549, and for IL-8 16HBE14o->hAECN>A549>hAECB). While 16HBE14o-cells produced equivalent levels of IL-6 and IL-8, A549, hAECN and hAECB cells produced up to 30-fold less IL-6 than IL-8.

When compared to controls, cells treated with single, binary and ternary mycotoxin mixtures usually released increased concentrations of IL-6 and IL-8 in a mycotoxin-dose dependent manner. Though, in response to DON, NIV and FX alone, hAECN cells poorly released IL-6 and A549 and 16HBE14o-cells poorly released IL-8. Globally, cytokine secretion was most obvious using mycotoxins at concentrations affecting the viability of 50% of cells, alone or in combination, for example IL-6 release by 16HBE14o- and IL-8 release by A549, 16HBE14o- and hAECB cells. However, low concentrations of NIV and FX alone or in combination with another mycotoxin were sufficient to stimulate IL-6 release by A549 and hAECB cells, and IL-8 release by hAECN cells. Only 16HBE14o-cells showed no effect of single mycotoxins at f_a_ 0.3. When comparing responses to low and high dose mycotoxins, there was a trend towards reduced IL-6 release by A549 cells exposed to NIV/FX and DON/NIV/FX and by hAECB cells exposed to DON/NV/FX and IL-8 release by hAECN cells exposed to NIV/FX and DON/NV/FX (Figure 4).

## 3. Discussion

Type B trichothecenes produced by *Fusarium* frequently contaminate dusts generated during grain harvesting and unloading, exposing grain workers to mycotoxins by inhalation [3,7,8,9,10]. While mycotoxins are thought to play a role in the development of airways disorders [35,36], a risk assessment is missing, among other reasons due to the lack of information on the mode of action of structurally related mycotoxins and on their combined effects on different levels of the human respiratory tract.

Here we compared the impact of DON, NIV and FX, alone and in combination, on the viability and capacity to release cytokines of immortalized cell lines and primary cell lines representative of different areas of the respiratory tract: nasal, bronchial and alveolar epithelium. We showed that FX is more toxic than NIV and DON for the four cell types tested, while DON is the least toxic toxin. This is in agreement with previous reports analyzing various immortalized cell lines including human U-937 histiocytic lymphoma [37], HL-60 promyelocytic leukemia [38] and Caco-2 colorectal adenocarcinoma [25,39], as well as the murine RAW 264.7 macrophage cell line [40] and insect SF-9 cells [41]. Strikingly, A549 cells were highly resistant to cytotoxic effects, and incubation periods of 48 h with mycotoxins were required to affect the viability/metabolic activity of 50% of cells. Accordingly, 24 h of exposure to as much as 25 μg/mL DON reduced the survival of A549 by only 30% [29]. However, a close look at the dose-cytotoxic effect curves revealed that primary cells were more resistant to low concentrations of DON, NIV and FX (i.e., concentrations < IC_50_), while they were more sensitive to high concentrations of single mycotoxins (i.e., concentrations > IC_50_).

A major observation of our work is that nasal and bronchial primary epithelial cells (hAECN and hAECB) were more sensitive to combined exposure to mycotoxins and behaved differently than immortalized alveolar (A549) and bronchial (16HBE14o-) epithelial cell lines in response to combined mycotoxin exposure. The interactions between DON, NIV and FX were more conservative for primary cells than immortalized cell lines. Except for hAECB cells, mycotoxins rarely acted in an additive manner, with synergistic or antagonistic interactions being frequently observed. Even for hAECB cells, DON and FX interacted in an antagonistic way and DON, NIV and FX in a synergistic way when used at low concentrations. Similar complex pictures have been reported previously for other cell types. For example, DON, NIV and FX had synergetic (DON plus NIV), additive (NIV plus FX) or antagonistic (DON plus FX) effects on the proliferation of IPEC-1 intestinal non-transformed cells [42]. Binary combinations of DON/FX and NIV/FX administered at low concentrations exhibited synergistic toxicity on Caco-2 cells, while DON-NIV-FX exhibited antagonistic effects [25].

The interactions of mycotoxins at low doses are particularly relevant, as those concentrations are closer to those met in occupational settings. At such concentrations (f_a_ 0.1), all combinations of mycotoxins acted in a synergistic way on hAECN cells, while more heterogeneous interactions were observed for bronchial and alveolar cells. Importantly, DON/NIV/FX acted synergistically on hAECB cells, while binary mycotoxin combinations acted either additively orantagonistically. Differences in cell response to mycotoxin exposure might be associated to differences in the prevalence of upper and lower respiratory symptoms in grain workers, which has been shown to vary according to the level of exposure to fungal spores contained in grain dusts [43]. Unfortunately, to our knowledge, there has been no report of *in vivo* models assessing the toxic impact of DON, NIV or FX delivered through bioaerosols. Supporting health risk concerns for occupational populations, DON reaches blood and tissues and stimulates inflammation more efficiently upon nasal delivery than oral delivery into mice [28].

Few studies explored the inflammatory response of respiratory cells to trichothecenes, and all were performed with immortalized cell lines with obvious limits of relevance. Trichothecenes interact with ribosomes to inhibit protein synthesis [20,44]. The following ribotoxic stress response activates the extracellular signal-regulated kinase-1/2 (ERK1/2), p38 and c-Jun N-terminal kinase 1 and 2 (JNK1/2) mitogen-activated protein kinase pathways that stimulate the transcription and production of cytokines in vitro and in vivo [45,46]. Accordingly, DON, NIV and FX stimulated IL-6 and IL-8 release by A549, 16HBE14o-, hAECB and hAECN cells, albeit at different levels. A recent study reported the dose-dependent release of IL-6 and IL-8 by A549 cells exposed to DON and the antagonistic effect of DON and summer or winter particular matter [30]. However, the range of DON concentrations (33.7 nM to 3.37 μM) tested was larger than the one screened here, which might explain why we did not observe a dose response effect with A549 cells. At least for IL-8, another explanation could be related to high basal expression of IL-8 by A549 cells, as previously observed [29], which makes it difficult to interpret the contribution of mycotoxin-induced IL-8 and IL-8 passive release from dying cells. Beside epithelial cells, macrophage cells might play a role in the lung inflammatory response to mycotoxins present in aerosols. Indeed, FX has been shown to increase TNF-α, IL-6 and IL-8 mRNA expression in U-937-derived macrophages [37]. However, DON, NIV, T-2 and HT-2 inhibited Toll-like receptor (TLR)/myeloid differentiation factor 88 (MyD88)/NF-κB activity in the THP-1 human macrophage cell line [47].

Concerning primary cells, increased IL-6 release was highest for hAECB cells, while IL-8 was induced in both hAECB and hAECN cells. Especially for hAECB cells, only the highest doses of mycotoxins induced IL-8 release. We cannot exclude that mycotoxins affecting cell viability by more than 50% would enhance cytokine release. However, such concentrations are unlikely to occur in occupational settings. Intriguingly, binary and ternary mycotoxin combinations did not significantly increase IL-6 and IL-8 release by primary cells when compared to single mycotoxins. A plausible explanation is that enhanced cytotoxicity by mycotoxin combinations impedes the capacity of cells to further increase cytokine production. This hypothesis is supported by the observations that DON stimulated cytokine production and immune functions at low doses, while it caused apoptosis leading to immunosuppression at high doses in human T lymphocytes and macrophages [48,49,50,51]. Nonetheless, DON and NIV were previously shown to synergize to increase the expression of proinflammatory cytokines in three-dimensional porcine jejunal explants [23]. Interestingly, ochratoxin A, a non-trichothecene mycotoxins occurring predominantly in cereals [52], induced IL-6 and IL-8 by primary nasal epithelial cells [53]. Thus, it would be important to analyze the effects of combinations of mycotoxins from different classes. Finally, we have previously shown that *Fusarium* spores potently stimulate innate immune cells to produce cytokines and chemokines [54], suggesting that exposure to both fungal particles and their derivative mycotoxins may participate to increase inflammatory processes. β glucans found in fungal cell walls are good candidates to stimulate inflammatory responses, as pointed out in experimental exposure studies in human and mice [55,56].

Overall, our data suggest that primary cells are more sensitive than immortalized cell lines to the exposure of low doses of mycotoxin in mixtures. Immortalized cell lines, while easy to handle, might not represent ideal models to accurately determine the mode of action of mycotoxins in vitro. In vitro experiments are however crucial to address the mode of action of mycotoxins with similar molecular structures and define their interactions when used in combination. This information should be taken into account when analysing the hazard of occupational exposure to mycotoxins by inhalation and its impact on airway cells, which, as suggested [23,24], cannot be deduced from experiments evaluating single mycotoxins.

## 4. Materials and Methods

### 4.1. Cell Culture

The A549 adenocarcinoma human alveolar basal epithelial cell line (CCL-185™, American type culture collection, Wesel, Germany) was maintained in RPMI 1640 medium (Invitrogen, Zug, Switzerland) supplemented with 10% fetal bovine serum (FCS, Sigma-Aldrich, St. Louis, MO, USA), 100 IU/mL penicillin and 100 µg/mL streptomycin (Invitrogen, Zug, Switzerland). The 16HBE14o-human bronchial epithelial cell line (kind gift of the Pulmonary service from CHUV, Lausanne, Switzerland) was maintained in modified Eagle’s medium (MEM, Invitrogen, Zug, Switzerland) supplemented with 10% FCS, 1% non-essential amino-acids (Invitrogen, Zug, Switzerland) and penicillin/streptomycin. Primary human airway epithelial cells isolated from bronchial (hAECB) and nasal (hAECN) biopsies were maintained in human airway epithelial cell medium (Epithelix, Plan-les-Ouates, Switzerland). Cells were cultured in 75 cm^2^ T-flasks at 37 °C and 5% CO_2_. Cells were detached by incubation for 5–15 min at 37 °C with 0.1 mg/mL trypsin-EDTA (Invitrogen, Zug, Switzerland), washed, enumerated and splited in 75 cm^2^ T-flasks or seeded in 96-well plates.

### 4.2. Regents and Chemicals

Deoxynivalenol (DON), nivalenol (NIV) and fusarenon X (FX) were purchased from Sigma (Saint-Louis, MO, USA). Stock solutions of mycotoxins were performed in methanol to reach 100 mM DON, 10 mM NIV and 100 mM FX. Working dilutions were prepared in cell culture media without FCS and antibiotics. Mycotoxin stock solutions as well as working dilutions were stored at −20 °C. Methanol at 0.2%, corresponding to the highest concentration reached upon cell exposure to mycotoxins, had no impact on cell viability and cytokine production. Methanol (LC-MS grade) was obtained from Carlo Erba Reagents (Val De Reuil, France).

### 4.3. Cell Exposure to Mycotoxins

Two × 10^5^ cells were seeded in 96-well plates in 200 µL of growth medium. After 18 h of incubation at 37 °C and 5% CO_2_, cells were exposed to mycotoxins alone or in combination for 24 h (16HBE14o-, hAECB and hAECN cells) or 48 h (A549 cells) to reach at least the half maximal inhibitory concentration (IC_50_). Negative controls were obtained by culturing cells with 0.2 to 2% methanol. At the end of the incubation period, 100 µL of supernatant were collected to quantify IL-6 and IL-8 by ELISA as described in Section 4.6, while cells were incubated with MTT to determine cytotoxicity following the protocol described in Section 4.4.

### 4.4. Cell Viability Assay

The MTT (3,(4,5-dimethylthiazol-2-yl) 2,5-diphenyltetrazolium bromide) cell proliferation assay (Sigma-Aldrich, Buchs, Switzerland) was used to assess the cytotoxic effect of mycotoxins. The assay is based on the conversion of MTT into a formazan product by mitochondrial succinate dehydrogenase. One hundred µL of 0.5 mg/mL MTT were added to each well and the plates were incubated for 1 h at 37 °C and 5% CO_2_ with the exception of hAECB, which where incubated for 3 h. A solution consisting of 10% SDS, 5 mM HCl and 66% isopropanol was used to lyse cells and dissolve formazan crystals. The absorbance was measured using a Synergy H1 microplate reader (BioTek Instruments, Luzern, Switzerland) at OD_570nm_ (experimental value) and OD_650nm_ (reference value). The percentage of viable cells was calculated as follows:

$$Cell\;viability\;\left(\%\right)=\;\frac{\left(Mean\;of\;OD\;mycotoxin\left(s\right)treated\;sample-Mean\;OD\;blank\right)}{\left(Mean\;OD\;negative-Mean\;OD\;blank\right)}\;\times \;100$$

### 4.5. Mycotoxin Cytotoxicity Assays and Data Analysis

Mycotoxins effects were tested at 0.2–58 µM DON, 0.1–21 µM NIV and 0.03–8.8 µM FX. Cell viability was determined and dose–response curves generated. The IC_50_ of mycotoxins was calculated with CompuSyn software version 3.01 (ComboSyn Inc., Paramus, NJ, USA) using the median-effect equation of the mass-action law:

(1) $$\frac{fa}{fu}={\left(\frac{D}{Dm}\right)}^{m}$$

where *D* is the dose of the mycotoxin, *f_a_* is the fraction affected by D (e.g., 1 − (percent viability)/100), and *f_u_* is the unaffected fraction (e.g., *f_u_* = 1 − *f_a_*). Dm is the median-effect dose (e.g., IC_50_) and *m* is the slope of the dose–effect curve. *m* = 1, *m* > 1 and *m* < 1 indicate hyperbolic, sigmoidal and flat sigmoidal dose–effect curves, respectively. Linear regressions of the median-effect curves of the mass action law were considered as acceptable if *r* > 0.9 [57].

The effects of the mycotoxin mixtures were determined for binary and ternary associations at IC_50_ ratio in order to enable similar toxicity for each mycotoxin. The cell viability values were used to define the type of interaction between the mycotoxins with CompuSyn software version 3.01. This software uses the combination index (CI) method derived from the median-effect principle to determine the interaction between two or three toxins over 10% to 90% *f_a_* according to the equation:

(2) (CI)xn= ∑j=1n(D)j(Dx)j

where *^n^*(*CI*)*_x_* is the *CI* for *n* mycotoxins at *x*% of inhibition, (*D*)*_j_* is the dose of n combined mycotoxins inducing *x*% of inhibition, (*Dx*)*_j_* is the dose of each mycotoxin alone inducing *x*% of inhibition. *CI* < 0.9, *CI* = 0.9–1.1 and *CI* > 1.1 reveal synergistic, additive and antagonistic effects. The *CI* values are expressed as the median of three independent experiments. Polygonographs depicting binary interactions for doses affecting the viability of 10, 30 and 50% of cells were plotted.

### 4.6. IL-6 and IL-8 Quantification by ELISA

The concentrations of IL-6 and IL-8 in cell culture supernatants were quantified using the Human IL-6 ELISA MAX Deluxe Set (BioLegend, San Diego, CA, USA) and the Human IL-8 ELISA Set (BD Biosciences, Franklin Lakes, NJ, USA) according to manufacturers’ recommendations. Each experiment was performed three times in triplicate.

### 4.7. Statistical Analyses

The values are median of three independent experiments performed in triplicate. The analysis of the dose–effect relationship of mycotoxin cytotoxicity and combination index (CI), 95% confidence intervals and dose reduction index calculation, isobologram plots and f_a_-CI plots for combined effects were all performed with Compusyn software version 3.0.1. For cytokine measurements, comparisons between groups were performed using the ANOVA F-test followed by a two-tailed unpaired Student’s *t*-test with Bonferroni correction. The level of *p* < 0.05 was considered statistically significant. Statistical analyses were conducted with PRISM (GraphPad, San Diego, CA, USA).

## Figures and Tables

**Table 1:** IC_50_ (in μM) of DON, NIV and FX for A549, 16HBE14o-, hAECN and hAECB cell viability inhibition assessed by MTT assay.

Mycotoxin	A549	16HBE14o-	hAECN	hAECB
DON	1.69 ± 0.49	2.2 ± 0.31	2.1 ± 0.88	1.97 ± 0.40
NIV	1.43 ± 0.37	1.52 ± 0.46	1.79 ± 0.45	1.58 ± 0.15
FX	0.27 ± 0.08	0.38 ± 0.06	0.61 ± 0.12	0.68 ± 0.14

Data represent median ± SD of three independent experiments performed in triplicate. The IC_50_ were determined following 24 h (16HBE14o-, hAECN and hAECB cells) or 48 h (A549 cells) of incubation.

**Table 2:** Mycotoxin concentrations (in μM) affecting the viability of 10% (f_a_ = 0.1) and 30% (f_a_ = 0.3) of cells.

Fraction Affected	Mycotoxin	A549	16HBE14o-	hAECN	hAECB
f_a_ = 0.1	DON	0.01	0.03	0.70	0.50
	NIV	0.07	0.10	0.60	0.50
	FX	0.02	0.01	0.20	0.23
f_a_ = 0.3	DON	0.25	0.28	1.40	1.00
	NIV	1.00	0.38	1.20	1.10
	FX	0.40	0.10	0.40	0.45

Data were deduced with CompuSyn for fractions affected (f_a_) of 0.1 and 0.3 (10% and 30% of cells) based on three independent experiments performed in triplicate. Cell viability was determined following 24 h (16HBE14o-, hAECN and hAECB cells) or 48 h (A549 cells) of incubation.
